# Presbyopia: Current Perspectives and Prospects for Patients

**DOI:** 10.1007/s44402-026-00083-7

**Published:** 2026-04-22

**Authors:** Leon N. Davies

**Affiliations:** School of Optometry, Aston University, Birmingham, UK

**Keywords:** Accommodation, Lens biomechanics, Patient reported outcomes, Presbyopia

## Abstract

Presbyopia, the ubiquitous loss of near vision function with age, has undergone significant reframing over the past decade. Indeed, in the last 10 years since *Ophthalmic & Physiological Optics’* last feature issue on presbyopia, research has progressed from viewing the refractive condition primarily as a decline in accommodative amplitude to recognising it as a multifactorial physiological process involving progressive lenticular stiffening, alterations in the capsule and zonule nexus and changes in optics. Advances in imaging and biomechanical assessment have deepened our understanding of the ageing crystalline lens and ciliary apparatus, while providing candidates for future clinical translation. Concurrent developments in correction and treatment have broadened the potential therapeutic landscape. Non-surgical approaches, particularly the emergence of pharmacological options including topical miotics and early-stage lens-softening agents, have marked the first major shift in presbyopia management in decades. Ongoing innovations in spectacle and contact lens designs have refined extended depth of focus and multifocal optics, while surgical strategies have continued to evolve. Despite these advances, consistent challenges remain in balancing near visual performance with quality of vision and in translating innovative laboratory and engineering innovations into stable, real-world patient benefits. In parallel, there has been a growing emphasis on patient-centred outcomes, reflecting an increased appreciation of the variability in visual demands, expectations and tolerance for compromises among presbyopic patients. Consequently, this narrative review synthesises some of the key developments related to presbyopia over the last 10 years, outlining future opportunities for restorative and personalised presbyopia care.

Key points

- Contemporary evidence shows that presbyopia arises from a multifactorial decline in the accommodative apparatus, with progressive lenticular stiffening, geometric changes and altered biomechanics.
- Over the past decade, presbyopia management has expanded to include pharmacological miotics, investigational lens softening agents, tuneable spectacle and contact lens technologies and surgical options, though long-term efficacy and durability remain variable.
- Future progress requires robust long-term clinical trials, improved in vivo biomechanical assessment tools, harmonised patient-reported outcome measures and a translational focus on real-world performance, durability and accessibility to ensure meaningful global impact.

## Introduction

With almost 2 billion people affected worldwide [1], presbyopia remains one of the most universal and consequential disorders of the healthy ageing eye, with the potential for profound personal, social and economic impacts [1–5]. A decade ago, *Ophthalmic & Physiological Optics* devoted a feature issue to the topic of presbyopia that brought together multidisciplinary insights, from objective measures of declining accommodative amplitude [6, 7] to the biomechanics of the crystalline lens and surrounding architecture [8, 9] and the functional consequences of correction strategies [10–12], setting out the research priorities that would help guide the field [13].

Since the 2016 *Ophthalmic & Physiological Optics* presbyopia feature issue, there has been continual and in some cases, rapid progress across three linked domains: deeper mechanistic understanding of presbyopia [14], emergent non-surgical [15, 16] and surgical [17, 18] treatment modalities and a stronger emphasis on patient-centred outcomes [19]. The historic view that presbyopia is simply loss of accommodative amplitude has matured into a multifactorial mechanistic picture [20, 21] in which increasing lenticular stiffness [22, 23], age-related changes in the lens capsule [24, 25] and zonular apparatus [8, 26] and optical factors such as changing depth of focus interact to produce the refractive condition described by Donders [27] and Duane [28] well over 100 years ago. These physiological foundations continue to motivate translational work seeking to restore dynamic focus or to replicate its functional effects.

Clinically, the past 10 years have seen the arrival of the first approved pharmacologic therapy for symptomatic presbyopia and a pipeline of topical agents with differing mechanisms of action. In 2021, the US Food and Drug Administration (FDA) approved a re-engineered pilocarpine formulation (pilocarpine HCl 1.25%, marketed as Vuity®, Allergan, Inc., an AbbVie company; abbvie.ie), the first topical correction modality specifically indicated to improve near vision in presbyopia [29], marking a pivotal shift toward drug-based management [30, 31]. More broadly, a rapid expansion of pharmacologic strategies (e.g., miotics, crystalline lens modifying agents and combination formulations) has emerged with growing randomised controlled data; however, questions remain about durability, side-effects and real-world patient selection.

Surgical and optical correction has also evolved [15, 17, 18, 32]. The last decade has seen refinement and proliferation of presbyopia correcting intraocular lenses, including trifocal and extended depth of focus (EDoF) designs. At the same time, many earlier mechanical approaches that aimed to restore true accommodation (e.g., some accommodating intraocular lens concepts) have produced mixed results, highlighting the ongoing technical and biological challenges of recreating dynamic accommodation in the ageing eye. Taken together, these developments argue for a reframing of presbyopia in clinical practice and research: from a single-solution problem (e.g., spectacles) to a spectrum of patient priorities that can be matched to a broadened armamentarium ranging from topical pharmacology and crystalline lens softening treatments to advanced wearable devices and intraocular solutions.

Consequently, this narrative review aims to synthesise some of the key findings relating to the definition, correction and treatment of presbyopia from the past decade, whilst identifying translational gaps and future priorities for patients. The aim is to provide clinicians and researchers with a physiologically grounded, patient-centred perspective on the contemporary understanding of presbyopia and the prospects for presbyopia correction in the future.

## A Contemporary Definition of Presbyopia

Over the last 10 years, considerable effort has concentrated on refining the definition of presbyopia [2, 3, 16]. This matters because estimates of prevalence and descriptions of presbyopia depend on both the diagnostic criteria and the measurement protocol. For example, detailed clinical assessments that correct distance refractive error and measure near-addition requirements may identify a different population from epidemiological surveys that classify presbyopia by unaided near visual acuity (UNVA). Therefore, where a definition sits on this continuum has a direct influence on who is considered presbyopic and on the subsequent interpretation of findings.

One clinical definition considers presbyopia as a symptomatic, age-related reduction in accommodative ability that remains after any distance refractive error has been optimally corrected [33]. This pathway generally requires a cycloplegic distance refraction to ensure full relaxation of accommodation, correction of any refractive error, assessment of unaided near vision at the patient’s habitual working distance and determination of the near addition required to restore satisfactory near acuity. Such a definition links the diagnosis to the patient’s optical status and task demands and will, therefore, capture individuals who rely on residual myopia for near tasks as presbyopic once distance correction is applied.

By contrast, field-orientated epidemiological studies frequently adopt simpler criteria: after confirming acceptable distance vision (occasionally using a pinhole), UNVA is measured and participants unable to meet a defined near acuity threshold are classified as presbyopic [34]. This expedient method systematically excludes uncorrected myopes from the presbyopia count, despite potentially equivalent reductions in accommodative amplitude. Between these extremes, hybrid definitions combine objective metrics (e.g., a specified dioptric shortfall or required near addition) with functional outcomes (e.g., the ability to perform specific near tasks), producing a range of prevalence estimates depending on thresholds and procedures.

Conceptual distinctions among definitions are also important. Some definitions focus narrowly on measurable near vision loss without reference to an individual’s visual needs [35, 36], risking misclassification of younger people with unrelated near impairment. Functional definitions, by contrast, constrain the diagnosis to instances where near clarity is insufficient for the individual’s requirements [37, 38]. Moreover, physiological definitions emphasise the age-related decline in the crystalline lens’s accommodative capacity as the underlying mechanism [39–41].

These contrasting perspectives are reconciled by a contemporary alternative definition of presbyopia, which states: presbyopia occurs when the physiologically normal age-related reduction in the eye’s focusing range reaches a point that, when optimally corrected for distance vision, the clarity of vision at near is insufficient to satisfy an individual’s requirements [2, 3]. This characterisation explicitly integrates the physiological basis (i.e., reduction in focusing range), the optical context (i.e., optimal distance correction) and the functional outcome (i.e., insufficient near clarity for the individual) and thus serves as a practical bridge between clinical precision and epidemiological applicability.

Consequently, to facilitate comparability and interpretation between clinical investigations, future studies relating to presbyopia should report working distance, acuity thresholds, correction status and whether near vision improvement with correction was required.

## Anatomical and Physiological Mechanisms of Presbyopia

Built on over a century of study, contemporary understanding of the ageing accommodative apparatus reveals progressive, inter-related structural and biomechanical alterations across the lifespan, with the crystalline lens, its surrounding zonules and the ciliary body/muscle nexus shaping the decline in amplitude and dynamic accommodation that characterises presbyopia.

### Crystalline Lens

Crystalline lens growth continues throughout life as new epithelial-derived fibres are laid down, producing a gradual increase in axial thickness of ~0.02–0.03 mm/year [42–44], whilst its equatorial diameter has been shown to remain unchanged [43] or increase [45] with age. Surface radii of curvature become steeper in the ageing eye, especially anteriorly [44, 46, 47] and the total protein content (e.g., α-, β- and γ-crystallins) of the lens increases [48, 49], leading to greater cellular compaction which, in turn, delivers a gradient of refractive index (GRIN) [50–52]. Modern GRIN models represent the crystalline lens as either a lamellar, shell-like structure [53] or as a smoother gradient across the lens [54] that changes in profile with age [52, 55–57].

A defining feature of the ageing crystalline lens is loss of compliance. Ex vivo studies document a greater than threefold increase in resistance to compression across the lifespan and show diminished focal change in response to simulated zonular tension in older eyes [58, 59]. Stiffness increases as a function of age in both the nucleus and cortex, though at different rates: in young lenses, the cortex is stiffer than the nucleus, whereas with ageing, the nucleus becomes the stiffer lenticular component [60, 61]. Representative ex vivo values illustrate this inversion with approximate log shear moduli of 1.5–2.0 Pa in a 20-year-old lens versus 3.2–4.2 Pa in a 70-year-old lens [60]. Increasing lenticular rigidity is widely regarded as the principal mechanical limitation to accommodation [23, 38, 62], although substantial variability in measurements and imperfect concordance with in vivo accommodative stimulus–response data, particularly before the fourth decade, indicate that stiffness alone does not tell the full story and that further in vivo studies are needed to elucidate fully the pathway to presbyopia.

### Zonular Apparatus

**Fig. 1:**
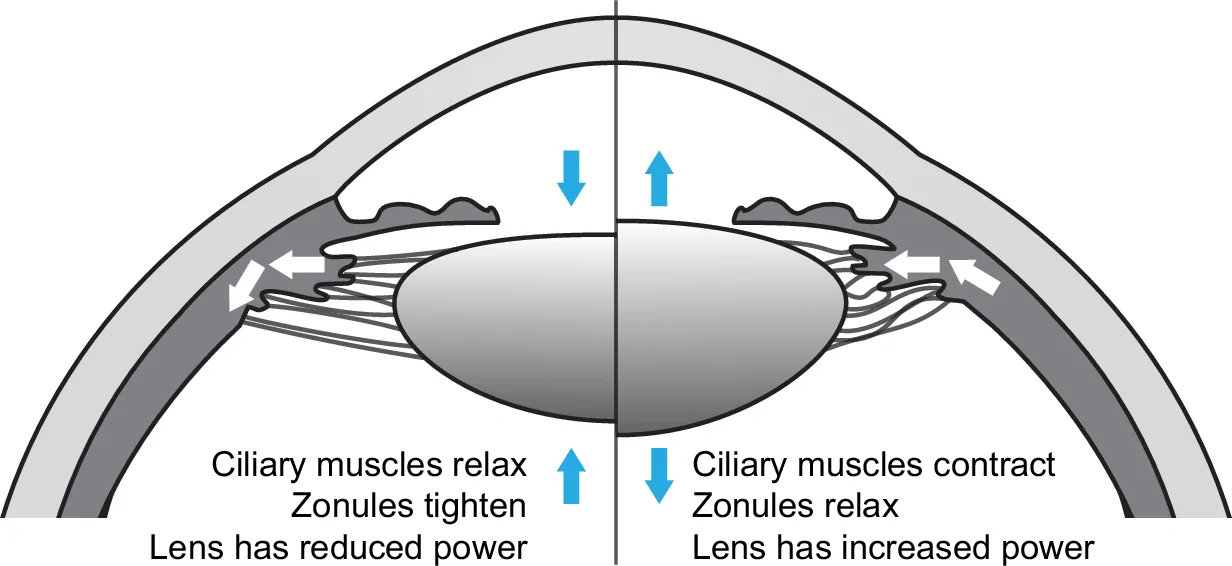
Principal components of accommodation. A schematic representation of lenticular and extralenticular changes during accommodation in the pre-presbyopic human eye. The left panel shows the relative position and morphology of the anterior eye in its relaxed state, whilst the right panel shows the eye during active accommodation with a forward and centripetal movement of the ciliary body on ciliary muscle contraction, leading to relaxation of the equatorial zonules and a corresponding increase in crystalline lens thickness and decrease in lens curvature in line with the Helmholtzian view of ocular accommodation [6, 45, 239–241].

Zonules form the connective framework linking the ciliary body to the lens capsule and transmit ciliary muscle forces that affect lenticular shape change during accommodation (Fig. 1) [63]. They consist of radial bundles arising from the vitreous framework and the ciliary processes, organised into anterior and posterior/vitreous subdivisions with distinct insertion patterns [8, 64, 65]. In vivo work has described a structure extending from the posterior vitreous zonule directly to the posterior lens equator that, together with posterior zonular fibres, may act to damp accommodation-related lens deformation in older eyes [8, 26, 66]. Whereas age-related geometric changes in the lens (e.g., axial growth and altered surface curvature) are hypothesised to alter the effective lever and vector relationships of zonular force transmission [67, 68], reducing the capacity of zonular tension changes to reshape the lens during accommodation [69, 70]. In vivo studies, therefore, suggest a progressive loss of zonular efficiency with age, but the magnitude and functional consequences of this effect merit further quantification.

### Ciliary Body and Muscle

The ciliary body, part of the uveal tract, contains the ciliary muscle, a multi-unit smooth muscle comprising longitudinal, radial and circular fibres, which mediates accommodation [71, 72]. The longitudinal fibres run from the scleral spur posteriorly, radial fibres bridge these orientations and circular fibres lie closest to the crystalline lens. Fibre-type distribution and mitochondrial content vary regionally and likely reflect differing contractile roles [73, 74]. Ciliary muscle contraction produces an antero-inferior and centripetal shift of ciliary muscle mass: longitudinal fibres contribute predominantly to the anterior displacement [71], while radial and circular fibres drive the inward movement and act as a local sphincter [69], with a temporal–nasal asymmetry that may assist lenticular axis alignment during convergence [75]. Ageing produces changes in ciliary muscle geometry and the circumlental space that, together with lens enlargement, reduce resting zonular tension and the effective lever for accommodation [76, 77].

Collectively, these structural and material changes in crystalline lens growth, progressive lenticular stiffening, evolving zonular architecture and shifts in ciliary muscle geometry, interact to reduce the amplitude and efficacy of accommodation. Whilst crystalline lens stiffening is central to accommodative decline, evidence supports a multifactorial model in which geometric, connective and muscular factors modulate how and by how much, lenticular shape can change with increasing age. Improved in vivo biomechanical techniques, measures and longitudinal studies are required to resolve remaining uncertainties and to inform future therapeutic strategies that aim either to restore lens flexibility or to exploit residual biomechanical capacity.

## Spectacle Correction

Spectacle correction for presbyopia remains foundational in clinical practice and continues to evolve along two complementary pathways: refinement of conventional lens optics [78, 79] and the use of electronically [80–83] or mechanically [84–87] tuneable spectacles. Conventional clinical options of single vision, segmented multifocal designs (e.g., bifocal, trifocal, quadrifocal) and progressive addition lenses, remain the clinical basis of contemporary presbyopia spectacle correction [15]. Most spectacle lens designs are the subject of ongoing optimisation to improve visual comfort and functional performance for modern visual demands [88–91].

### Potential Future Directions for Presbyopia-correcting Spectacles

Future advances in progressive addition lens technology are likely to emphasise optical optimisation for real-world tasks [92, 93]. These include further reduction of unwanted astigmatism [94, 95], improved intermediate vision zones tailored to common device viewing distances [96, 97] and expanded consideration of pupil dynamics [98] and viewing posture [93, 99, 100] in lens design. The need for device personalisation is a major theme [101–103]. Such personalised optics aim to maximise usable fields of view and minimise adaptation time for patients with diverse occupational and lifestyle visual demands [104, 105].

Parallel to incremental optical improvements, more radical attempts to restore a form of dynamic focus change to spectacle wearers have been pursued. Tuneable-power spectacle lenses seek to reproduce an accommodation-type change of focal power, either by mechanical rearrangement of lens elements (e.g., Alvarez-type variable lenses) [106] or by utilising electrically responsive materials (e.g., liquid-crystal stacks) [107]. Hitherto, clinical adoption of these designs is mixed, with some liquid-crystal spectacles failing to achieve sustained market traction for a variety of optical, manufacturing and user acceptance reasons [108], whereas Alvarez-based self-adjusting lenses have been brought to market and evaluated both for presbyopic correction and as low-cost, self-refraction aids in paediatric settings with limited refractive services [109].

Spectacle management of presbyopia over the coming years will, therefore, likely be characterised by two complementary trends. First, improved progressive addition lenses and multifocal optics, personalised to the wearer’s behaviour and frame geometry, will incrementally enhance function for a wide population. Second, tuneable spectacle technologies offer a disruptive potential to approximate dynamic accommodation, but they require further optimisation of ergonomics, optical quality and affordability before widespread clinical uptake. Rigorous clinical trials that examine binocular performance, adaptation, long-term tolerability and real-world utility will be essential to determine which, if any, of these innovative approaches will translate into meaningful benefits for patients.

## Contact Lens Innovations

Contact lenses remain a key clinical tool for the correction of presbyopia, with contemporary options including monovision [110], simultaneous soft contact lenses [111], rigid translating [108] and rigid simultaneous [112] designs. Whilst the current range of options available to eye care practitioners is wide across material platforms, in recent years, a clear trend towards the use of soft contact lenses has emerged. Indeed, based on data between 2000 and 2023 from presbyopes aged ≥45 years living in 20 countries across the world, multifocal and monovision soft daily wear lens prescribing has more than doubled to over 60% of standard soft daily wear lens fits; although it is worth noting that this trend is not seen in all populations [110]. Further, the relative proportion of fits to presbyopes is heterogeneous, with 51% of presbyopic patients wearing multifocal designs, 10% in monovision and a large minority (39%) fitted with non-presbyopia correcting modalities [110], with subsequent near vision correction most likely provided by single vision spectacles.

Therefore, much focus is placed on multifocal lens designs, with several optical techniques available to eye care practitioners to facilitate vision at a range of distances (e.g., diffractive [113], simultaneous imaging [114] and extended depth of focus [115]). Whilst many of the age-dependent factors associated with the ageing eye (e.g., reduced tear production and stability [116]) are largely overcome with advances in lens design and materials, intrinsic factors such as pupil miosis [117] and crystalline lens transparency [46] changes in the ageing eye reduce retinal luminance by over 60% by the age of 60 years [118]. Coupled with parallel increasing ocular aberrations [119] and internal light scatter [120], retinal image quality of the presbyopic and ageing eye degrades over time [121]. Consequently, future contact lens designs and similar optical devices should seek to limit further degradation of retinal image quality through changes in retinal illuminance and/or image contrast.

### Potential Future Directions for Presbyopia-correcting Contact Lens

A range of novel accommodating or focus-tuneable contact lens designs have been proposed with the goal of replicating the pre-presbyopic, dynamic focusing ability of the young eye [122]. These approaches can broadly be grouped into mechanically actuated, fluidic, electroactive/electronic and liquid-crystal solutions, but in practice, many designs combine elements from more than one category [15]. However, designing such lenses presents several challenges. Indeed, to function effectively, these contact lens designs must detect gaze and viewing distance and reversibly adjust optical power whilst preserving comfort, ocular health and visual quality. Unlike spectacle lenses, where eye rotation permits visual axis alignment with near or distance zones, contact lenses move with the eye and, therefore, must locate gaze and distance and change focus in real time. Practically, an effective system should change focus with viewing direction to deliver usable distance vision and approximately an additional +2.00 D for near tasks [15, 123].

Mechanically driven concepts exploit eyelid pressure or engineered structural elements to alter lens geometry and thus dioptric power. In one class of design, the anterior surface of a rigid lens is designed so that lid interaction reshapes the optic to increase power for intermediate or near tasks [124]. Fluidic variants embed a pupil-aligned chamber connected to a peripheral reservoir; an on-lens actuator transfers fluid between reservoir and chamber in response to a gaze sensor and control signal, producing a centre-aperture power change from distance to near [125]. Electromechanical pumps have also been proposed to move fluid centrally on command, accomplishing a similar effect to the mechanically driven approaches [125].

Electronic contact lenses, sometimes termed smart contact lenses [15], are designed to integrate microelectronics to sense eye position and drive optical actuators. Here, capacitive corneal gaze sensors have been demonstrated to infer gaze direction from capacitance changes [126]. For such systems to have future practical application, they will require a compact power source and a form of telemetry (i.e., a battery and antenna), reliable control electronics and a means of safely housing these components within a soft, biocompatible material. Several patents describe architectures in which integrated chips, sensors, actuators and power management could be embedded within the contact lens matrix [126–128].

Electroactive materials, sometimes termed accommodation actuators, offer an alternative route to presbyopia correction. Here, electrically driven elements are designed to change the contact lens shape or refractive index of a polymer gel [129], in response to a control signal (e.g., Bluetooth® Low Energy, Radio Frequency Identification or Near Field Communication), producing rapid, reversible power shifts [130, 131]. These elements may be placed within the optic zone or dispersed through the bulk of the lens material coupled to a gaze detection system, controller and power supply [132]. Similarly, liquid-crystal-based optics [107] reorient liquid-crystal molecules sandwiched between transparent electrodes to change refractive index and optical power [133–135]. The technology could also function as a dynamic pinhole to increase depth of focus [15].

Although the engineering foundations are promising, most accommodating contact lens concepts remain at the patent or prototype stage. Key translational hurdles include providing safe, long-term power and wireless communication, ensuring on-lens electronics and actuators tolerate the ocular surface environment, preventing deposition and maintaining wettability and demonstrating that dynamic optics yield clinically meaningful, comfortable vision in daily life. Rigorous clinical trials will be required to assess safety, efficacy and durability before any of these technologies can be adopted for routine presbyopia management, but the diverse and creative approaches now being pursued suggest substantial industry and academic interest that is likely to drive further advances in this area.

## Non-surgical Approaches to Presbyopia Correction: A Decade of Development

In pursuit of what is sometimes referred to as the holy grail of vision correction [136, 137], the most fundamental approach to ameliorate the symptoms manifest by presbyopia is to modify the underlying biological processes responsible for its development. Whilst it is clear these age-related processes arise predominantly within the crystalline lens [60], other components of the accommodative apparatus may also contribute [8, 64]. Hitherto, there is no conclusive evidence that proposed early interventions can meaningfully slow the age-related decline in accommodation, even though the presence of factors such as dietary antioxidants, e.g., α-carotene and β-carotene [138], riboflavin [139], vitamin A [140] and zeaxanthin [141] are known to be associated inversely with the formation of early lens opacities [142].

Data suggest that certain environmental exposures, including elevated ambient temperatures [143–145], urban versus rural location [146–148] or possibly prolonged sunlight exposure [149, 150], accelerate the onset of presbyopia. These findings have led to speculation that protective strategies might offer some benefit to arrest presbyopia development; however, further research is required. Moreover, given that presbyopia typically emerges in mid-life [151], individuals are likely to live for decades with the condition, particularly in developed nations. Consequently, any treatment or preventative approach should delay presbyopia onset by several years to achieve meaningful patient benefit. That said, recent years have seen growing interest in a battery of innovative non-surgical approaches to presbyopia correction [15, 32].

### Pharmacological Approaches

Over the last decade, pharmacological approaches have emerged as a leading clinical strategy for presbyopia correction [152–154]. Indeed, a variety of formulations are currently commercially available or currently under investigation [16]. Of these, two strategies have gained particular prominence: agents that constrict the pupil to increase depth of focus, often with a potential secondary myopic shift [155] and compounds intended to restore dynamic accommodation by softening the ageing crystalline lens [32].

### Pupil Constriction

Several investigational regimens target pupil size and, in some instances, transiently stimulate the ciliary muscle, typically up to ~8 h. A large retrospective, non-randomised study evaluating the topical application of pilocarpine plus preservative-free diclofenac between January 2011 and June 2018 reported a trend toward near-vision spectacle independence [156]. Among 910 participants aged 40–59 years, baseline Jaeger UNVA improved from 4.74 (SD 1.53) to 1.36 (SD 0.48) over the study period, while binocular uncorrected distance visual acuity (UDVA) remained essentially unchanged (baseline 0.00 logMAR; at 8 years 0.03 logMAR). Reported adverse effects, such as reduced light perception, headache, ocular surface dryness and dizziness, tended to resolve in those who continued treatment [156].

Novel topical preparations have produced similarly encouraging short-term gains. In 177 presbyopes (aged 41–65 years), a single-product evaluation of FOV tears (a combination of pilocarpine and phenylephrine) demonstrated a significant improvement in UNVA from 0.35 to 0.16 logMAR at 2 h post-instillation, although nine participants showed no benefit and ~14 participants (12%) reported headache. Younger participants gained more lines, implying an additional mechanism beyond miosis, such as a small myopic shift in refraction [157]. A subsequent report found statistically significant UNVA improvements across cohorts that included prior LASIK, monovision or PresbyLASIK (i.e., corneal refractive surgery to create a multifocal corneal surface analogous to a multifocal contact lens) patients and nonsurgical controls [158]. Further research is necessary to evaluate the long-term effect of the agent and associated risks.

Importantly, in 2021, a topical formulation of pilocarpine HCl 1.25% (Vuity®; Allergan, Inc., an AbbVie company; abbvie.ie) became the first FDA-approved pharmacological treatment for presbyopia [29]. A vehicle-controlled, participant- and investigator-masked, randomised, phase 3 clinical study (GEMINI 1) enroled 323 individuals with presbyopia, aged 40–55 years, between December 2018 and October 2019 [30]. GEMINI 1 demonstrated notable enhancements in near vision at day 30 over the control group, with participants achieving an increase of ≥3 lines in mesopic, high-contrast, binocular distance corrected near visual acuity (DCNVA), while maintaining distance vision with no loss beyond 5 letters. The study also indicated that the onset of effect was at 15 min and lasted for up to 6 h [30]. In terms of reported adverse events, the most common symptom reported in the active group was headache (14.1%, 87% of which were considered mild with no treatment required), followed by visual impairment (4.3%).

To address the paucity of data on night vision, a subsequent study determined the impact of Vuity® on night driving performance [159]. Here, overall driving performance at night in real-world conditions was inferior at the end of a 7–14-day intervention period 1-h post-instillation with pilocarpine 1.25% compared with a placebo. The measured vision loss was between 0.2% and 13.3% versus the placebo condition. However, this was within the American National Standards Institute’s (ANSI) non-inferiority margin of 25% for assessing night-driving performance of multifocal versus monofocal intraocular lenses [160]. These data are important to assist eye care practitioners in considering prescribing pilocarpine 1.25% to adults who seek treatment of presbyopia symptoms and drive at night.

The advent of Vuity® will likely catalyse future research in this area, promoting further pharmacological treatments and regimen refinements. Whilst data suggest single dosing of pilocarpine 1.25% provides measurable improvements in near vision for up to 6 h, further work is indicated to quantify the impact of multi-dosing to provide adequate near vision throughout the day. Furthermore, although pupil miosis effectively increases depth of focus with modest impact on peripheral optics [121, 161], its effects on retinal illuminance [162] and functional near acuity [163] under varying luminance and contrast conditions warrant further investigation. The real game-changer will be the drug that is supported by direct evidence for drug-induced restoration or enhancement of true dioptric accommodation, resulting in a measurable change in ocular power.

### Crystalline Lens Softening

Efforts to develop a crystalline lens softening strategy for presbyopia treatment aim to reverse age-related lenticular stiffening by cleaving protein disulfide bonds. Formulations based on lipoic acid and choline esters are proposed to reduce intralenticular disulphides and thereby increase lens elasticity [164]. Early clinical work has produced encouraging but mixed results.

An initial clinical evaluation of EV06 (UNR844) reported improved DCNVA after a 90-day dosing regimen (twice daily after an initial week) [165]. A small follow-up cohort, comprising 34 treated versus 18 control participants, showed that visual gains persisted for ~6 months after cessation of treatment [166]. A prospective, multicentre study of the lipoic-acid choline ester UNR844 (1.5%) in 75 presbyopes likewise showed an acceptable safety profile and a statistically greater mean improvement in treated eye DCNVA over 91 days (mean logMAR change −0.159 versus −0.079 for placebo). Bilateral outcomes favoured UNR844, with 53.1% versus 21.7% of participants gaining ≥10 letters. Importantly, these gains were reported to persist at 5 and 7 months post-treatment [167]. However, a larger Phase-2 study of 124 participants across both the study and placebo arms in 45–55-year-olds, failed to demonstrate a significant binocular DCNVA advantage for UNR844-CI versus placebo (mean letters 6.1 versus 4.5, *p* = 0.18), nor a significant difference in participants achieving ≥75 Early Treatment of Diabetic Retinopathy Study (ETDRS) letters at month 3 (25.0% versus 15.8%, *p* = 0.28) [168].

Collectively, crystalline lens softening therapies represent a mechanistically attractive, potentially restorative alternative to optical correction. That said, current evidence is limited. Adequately powered, longer-duration randomised trials are, therefore, required to define the magnitude, durability and clinical significance of near vision gains and to characterise long-term safety before these agents can be adopted as conventional presbyopia treatments.

## Surgical and Interventional Approaches

### Intraocular Lenses

Whilst standard monofocal intraocular lenses have historically been the most widely implanted intraocular lens type for typical cataract or refractive lens exchange patients [169–171], data from 2024 suggest UK refractive surgeons are now opting for more presbyopia correcting intraocular lenses, with EDoF lenses (30%), monofocals (20%) and trifocals (20%) the most popular lens choices [172]. The most cited reason for their choice of each lens modality was better overall visual outcomes (88.9%), fewer unwanted symptoms (66.7%) and best spectacle independence (66.7%), respectively [172].

Refractive multifocal intraocular lenses typically employ either concentric or segmented optical strategies. Concentric refractive designs incorporate annular zones of additional power within the optic so that light traversing different rings is directed to nearer focal planes, superimposed on the optic power for distance. Conversely, segmented intraocular lenses are rotationally asymmetric and localise the near zone to a discrete sector of the optic, such that the proportion of the near segment captured by the pupil determines the amount of light directed to near vision. As segmented intraocular lenses depend on precise alignment, decentration can degrade image quality [173, 174]; however, when adequately centred, they generate relatively low rates of glare and halo [175], with studies reporting good visual outcomes and patient satisfaction [176, 177].

Diffraction-based multifocal intraocular lenses use phase steps to diffract incoming light into distinct focal planes, with the spacing of successive diffractive ring edges determining the effective near add [178]. When the diffractive profile spans the central optic (i.e., fully diffractive designs), the allocation of light to each focus is relatively insensitive to pupil size. Conversely, lenses that confine the diffractive structure to a subregion of the optic (i.e., partially diffractive designs) show a greater dependence of near performance on pupil diameter [179].

EDoF intraocular lenses are designed to produce a single, lengthened focal region rather than discrete foci, thereby increasing functional depth of focus [180]. By spreading light less discretely across multiple planes than conventional multifocal designs, EDoF optics tend to mitigate some loss of contrast and reduce dysphotopsia [181], whilst delivering good distance and intermediate visual acuity [182]. Consequently, EDoF lenses are recommended for patients whose near-vision requirements are intermittent or of lower acuity demand [183–185], with reading spectacles advised for concentrated near tasks [186].

For intraocular lenses to be truly accommodative, they should demonstrate a measurable dioptric change in vivo [171]. Most contemporary accommodative concepts rely on preserved ciliary muscle contractility, transmitting forces via the zonules and capsular bag to alter lens position or morphology [2, 18]. Flexible haptic intraocular lenses, designed to move anteriorly under ciliary muscle forces and thereby increase optical power, produce reports of improved intermediate and near acuity [187–190]; however, objective measures of accommodative amplitude [191] and lens position [192] are generally small and any early subjective near vision benefits may diminish with increasing levels of capsular fibrosis [193]. Indeed, the extent to which such lens designs produce a durable dioptric change on accommodation is debatable [21]. Designs aiming for larger dynamic ranges during accommodation include dual-optic systems [194–196]. Here, a high-plus anterior element is separated from a negative posterior optic. One study demonstrated greater effective accommodative movement of 2.4–2.5 D/mm compared to 1.4–1.5 D/mm of movement for a mono-optic design [197]; however, few studies have been published on these designs over the last 10 years.

After more than two decades of innovation, a genuinely accommodative intraocular lens that produces a robust, sustained and clinically useful change in dioptric power remains elusive. Capsular fibrosis and other biological factors continue to blunt the putative dynamic behaviour of many designs and evidence suggests that much of the near benefit seen with nominally accommodative implants may reflect pseudo-accommodation, perhaps via changes to the aberration profile, rather than any actual restoration of accommodation.

### Corneal-Based Surgical Approaches

PresbyLASIK uses excimer ablation to create corneal multifocality [198], improving near vision at the cost of increased higher-order aberrations. Approaches include central [199], peripheral [200] and blended [201] profiles, each balancing near gains against degraded distance acuity. Indeed, despite generally good optical outcomes for patients [202], many report dissatisfaction owing to compromised distance vision despite near improvement, suggesting monocular treatments are more likely to be tolerated by those who require excellent distance vision [202, 203].

Corneal inlays improve near and intermediate acuity reliably with relatively little detriment to distance vision. A systematic review of 18 studies (2,724 eyes) reported 78.5% achieving UNVA ≥ 6/9.6 (~0.20 logMAR) and 90.5% UDVA ≥ 6/7.5 (~0.10 logMAR) [204]. Early reports for shape-changing and multifocal inlays were promising, but longer follow-up exposed safety issues. For example, the previously FDA-approved, but no longer available, Raindrop Near Vision implant (ReVision Optics) showed high early near gains yet a 5-year corneal haze incidence that prompted market withdrawal [36]. Conversely, devices such as the KAMRA^TM^ inlay (AcuFocus Inc., a Bausch + Lomb company; ir.bausch.com) have delivered durable near vision [205–207]. Notwithstanding these benefits, 10-year explantation rates of 8.2% have been noted [208], as inlays can induce refractive shifts [209] and reduced contrast sensitivity [210]. Consequently, appropriate patient selection is critical [211].

In the search for a more corneal biocompatible material, allogeneic corneal inlays, using donor small incision lenticule extraction (SMILE) lenticules [212], have been developed to avoid synthetic inlay complications. Femtosecond-assisted small incision allogeneic lenticule implantation, including the PrEsbyopic allogenic refractive lenticule (PEARL) and the TransForm™ corneal allograft (Allotex; us.allotex.com), has shown improved near vision with acceptable safety up to 3 [213] and 4 [214] years of follow-up.

### Scleral and Extraocular Procedures

Scleral expansion bands are intended to widen the circumlental space between the ciliary body and the crystalline lens and thereby restore zonular tension [215], a strategy grounded in the somewhat controversial [216] model that age-related equatorial lens growth reduces the ciliary muscle-to-crystalline lens distance and limits zonular force [217, 218]. Reported clinical outcomes have been variable and generally modest. Two-year trial data (NCT02374671) of the VisAbility™ Micro Insert System (Refocus Group Inc.; refocus-group.com), with 360 baseline participants, revealed 84% of 344 analysed eyes achieved DCNVA of 6/12 at 40 cm with a ≥10-letter gain at 24 months. Anterior segment ischaemia was reported in some cases, although most patients experienced no serious ocular adverse events [219]. A longer-term follow-up study (NCT03811249) at 36, 48 and 60 months with 287 baseline participants, revealed 83% of 193 primary eyes achieved DCNVA of at least 6/12 by 60 months post-surgery [220].

Alternatively, the proposed technique of laser scleral microporation aims to increase scleral compliance and reduce ocular rigidity by creating arrays of laser-generated micropores in the sclera overlying key accommodative structures within five anatomical zones, including the scleral spur and the three ciliary muscle fibre groups [221, 222]. These fractional defects are proposed to reduce cross-linking, produce local tissue softening and permit greater scleral deformation in response to ciliary muscle forces, thereby lowering resistance during accommodation and improving biomechanical efficiency.

## Patient-centred Outcomes and Quality of Life

Presbyopia imposes a substantial and globally distributed burden on visual function and quality of life [4, 223–225]. To capture patient impact and to evaluate the effectiveness of interventions from the patient perspective, a variety of patient-reported outcome (PRO) instruments have been developed, both to inform clinical research and to support regulatory and commercial claims [226–229]. A review published in 2020 [230] highlighted a notable paucity of instruments developed specifically for presbyopia. Indeed, from the range of PROs used to assess vision-related outcomes in presbyopia or similar conditions, only the near activity visual questionnaire (NAVQ) [228] was presbyopia-specific [230]. In order to align the NAVQ with technological (e.g., smartphone use) and lifestyle changes since its original development in a wholly phakic cohort, the NAVQ-P was developed and evaluated in accordance with the US FDA PRO guidance with the aim of improving its applicability for contemporary presbyopia trials and product labelling [231, 232]. Additional single-item instruments of near vision correction independence (NVCI), correction preference (NVCP) and vision satisfaction (NVS) were also developed [231].

As alternatives to fixed questionnaires, item-banking approaches coupled with computer-adaptive testing (CAT) have been advanced [229, 233, 234]. Using calibrated item banks, CAT delivers individually tailored item sets (typically only a few items) and can estimate refractive error-related quality of life domains efficiently via staircase-type algorithms [233]. Item banking with CAT has been proposed in the literature as a route to replace traditional PROs in both research and routine care because of its measurement precision and reduced respondent burden [235].

All questionnaire-based methods, however, remain dependent on participants’ recollection and subjective interpretation of visual experiences, which can introduce bias. To complement question-based assessment, the Multifocal Acceptance Score (MAS-2EV) has been developed to elicit direct judgements of perceived visual quality using a series of digitised images that represent common daily tasks, together with a near stereoacuity test [236]. Early work suggests the MAS-2EV may be useful for comparing presbyopic correction options at the individual level [237, 238]. Further studies are suggested to determine its performance against validated questionnaire instruments and its potential role alongside CAT-based measures.

## Conclusion

The past decade has witnessed substantial and at times rapid progress in both our understanding and management of presbyopia. Mechanistic work has moved the field to a multifactorial understanding in which progressive lenticular stiffening, changes to lens geometry, zonular and capsular alterations and uveal biomechanics all contribute to diminished dynamic focusing and, therefore, presbyopia.

Clinically, the portfolio of corrective options available to eye care practitioners and their patients has broadened markedly. Non-surgical approaches, most notably pupil-modulating pharmacotherapies, have transitioned from concept to clinical trials and, in one instance, regulatory approval. Spectacle and contact lens technologies continue to be refined and personalised, whilst intraocular devices have developed. Concurrently, attempts to restore sustained accommodation to the ageing eye have highlighted persistent biological and/or engineering barriers, with lenticular capsular fibrosis, long-term device durability and tear-film compatibility remaining important unresolved challenges for a range of putative interventions.

For the presbyopia research and development community, future priorities are clear. First, adequately powered, long-duration randomised trials of new interventions are needed to characterise effectiveness, durability and safety across diverse populations. Second, additional investment in reliable in vivo biomechanical devices and restorative therapies could provide a step change in presbyopia care. Third, harmonised PRO instruments are essential to compare interventions directly and to align patient priorities. Fourth, translational work should emphasise durability and real-world ergonomics, not only acute efficacy and safety and must address affordability and equity if novel presbyopia therapies are to achieve global public health impact.

The decade since *Ophthalmic & Physiological Optics’s* presbyopia feature issue has, therefore, delivered both deeper physiological understanding and a broader approach to presbyopia correction and treatment. The opportunity now is to capitalise on these advances to improve patient choice and deliver long-term positive clinical outcomes for presbyopes.

## Data Availability

No datasets were generated or analysed during the current study.
